# Improving inappropriate medication and information transfer at hospital discharge: study protocol for a cluster RCT

**DOI:** 10.1186/s13012-018-0839-1

**Published:** 2018-12-27

**Authors:** Thomas Grischott, Stefan Zechmann, Yael Rachamin, Stefan Markun, Corinne Chmiel, Oliver Senn, Thomas Rosemann, Nicolas Rodondi, Stefan Neuner-Jehle

**Affiliations:** 1 Institute of Primary Care (IHAMZ), University and University Hospital of Zurich, Pestalozzistrasse 24, CH-8091 Zurich, Switzerland; 2 Institute of Primary Health Care (BIHAM), University of Bern, Bern, Switzerland; 3 Department of General Internal Medicine, Inselspital, Bern University Hospital, Bern, Switzerland

**Keywords:** Multimorbidity, Polypharmacy, Hospital discharge, Medication review, Deprescribing, Potentially inappropriate medication, Patient priorities, Cluster-randomised controlled trial

## Abstract

**Background:**

Inappropriate medication and polypharmacy increase morbidity, hospitalisation rate, costs and mortality in multimorbid patients. At hospital discharge of elderly patients, polypharmacy is often even more pronounced than at admission. However, the optimal discharge strategy in view of sustained medication appropriateness remains unclear. In particular, unreflectingly switching back to the pre-hospitalisation medication must be avoided. Therefore, both the patients and the follow-up physicians should be involved in the discharge process. In this study, we aim to test whether a brief medication review which takes the patients’ priorities into account, combined with a standardised communication strategy at hospital discharge, leads to sustained medication appropriateness and extends readmission times among elderly multimorbid patients.

**Methods:**

The study is designed as a two-armed, double-blinded, cluster-randomised trial, involving 42 senior hospital physicians (HPs) with their junior HPs and 2100 multimorbid patients aged 60 years or older.

Using a randomised minimisation strategy, senior HPs will be assigned to either intervention or control group. Following instructions of the study team, the senior HPs in the intervention group will teach their junior HPs how to integrate a simple medication review tool combined with a defined communication strategy into their ward’s discharge procedure. The untrained HPs in the control group will provide data on usual care, and their patients will be discharged following usual local routines.

Primary outcome is the time until readmission within 6 months after discharge, and secondary outcomes cover readmission rates, number of emergency and GP visits, classes and numbers of drugs prescribed, proportions of potentially inappropriate medications, and the patients’ quality of life after discharge. Additionally, the characteristics of both the HPs as well as the patients will be collected before the intervention. Process evaluation outcomes will be assessed parallel to the ongoing core study using qualitative research methods.

**Discussion:**

So far, interventions to reduce polypharmacy are still scarce at the crucial interface between HPs and GPs. To our knowledge, this trial is the first to analyse the combination of a brief deprescribing intervention with a standardised communication strategy at hospital discharge and in the early post-discharge period.

**Trial registration:**

ISRCTN, ISRCTN18427377. Registered 11 January 2018

**Electronic supplementary material:**

The online version of this article (10.1186/s13012-018-0839-1) contains supplementary material, which is available to authorized users.

## Background

Polypharmacy, particularly among multimorbid older patients, is associated with increased risks of adverse drug reactions and interactions, prescription and intake errors and low patient adherence [1–3], resulting in higher morbidity, hospitalisation rates, costs and mortality in affected patients [4–6]. Along with rising levels of mulitmorbidity, the prevalence of and therefore the importance to manage polypharmacy have significantly increased over the last years [7], and related recommendations and guidelines have emerged [8, 9].

Hospitalisation is strongly associated with polypharmacy, with the number of drugs at discharge being considerably higher than at the time of admission [10]. Furthermore, a high number of drugs at hospital discharge has been shown to be, independent of relevant comorbidities and cognitive status, a predictor for early readmission in older patients [11]. Nonetheless, interventions to reduce polypharmacy—which exist in variable grades of complexity, feasibility and dissemination—have so far mainly been adopted by specialists like geriatricians or pharmacologists but are not widely used at the crucial interface between hospital physicians (HPs) and general practitioners (GPs) [12–18].

This is surprising given that suitable discharge interventions have the potential to considerably reduce readmission rates and extend readmission times: studies have shown reductions of 30-day readmission rates between 15% and 50% [19, 20], and a recent systematic Cochrane review demonstrated a relative risk reduction of 13% (RR 0.87; 95% CI 0.79–97) for readmission after planned discharge interventions [21]. Time to readmission could be extended by one third (from 12 to 18 days) in a frail population of older adults (≥ 60 years) by a transitional care program [22].

However, in order to persistently reduce polypharmacy among discharged patients, it is not sufficient to optimise medication plans at discharge. Unreflectively switching back to pre-hospitalisation medication schemes has been identified as a common pitfall to be avoided for a sustained effect [23]. Consensus between the hospital and follow-up physicians with regard to the discharge medication leads to higher adoption rates of the medication plans in the post-discharge period, compared to unidirectional communication [24]. Therefore, it is of utmost importance to involve both the patients and their GPs in the prescribing/deprescribing decisions at discharge [25].

None of the studies mentioned above—and to our knowledge no other trial either—analysed the effects of a discharge strategy which incorporates both crucial aspects of deprescribing and collaborative communication between HP and GP at hospital discharge. This paper describes the protocol for a randomised controlled study designed to fill this gap.

### Trial objectives

The aim of this study is to investigate the benefits of reducing polypharmacy among discharged multimorbid hospital patients by means of an improved discharge procedure combining a brief deprescribing intervention and a standardised communication strategy. In particular, we aim at analysing the impact of the new procedure on readmission rates and times, the number of drugs as well as the proportion of potentially inappropriate medications (PIMs) at discharge, and on the discharged patients’ quality of life (QoL) within a 6 month follow-up.

Secondary objectives are to explore determinants of the new procedure’s implementation into the daily routine and to estimate potential cost savings, in order to build the empirical basis for its future dissemination as a “best practice” model among Swiss hospital wards and GPs.

### Study hypothesis

We hypothesise that a simple medication review tool in combination with a defined communication strategy at hospital discharge (intervention group) extends time to hospital readmission compared to usual care (control group) and improves the patients’ health outcomes and QoL in the post-discharge period.

## Methods/design

### Study design and setting

**Fig. 1:**
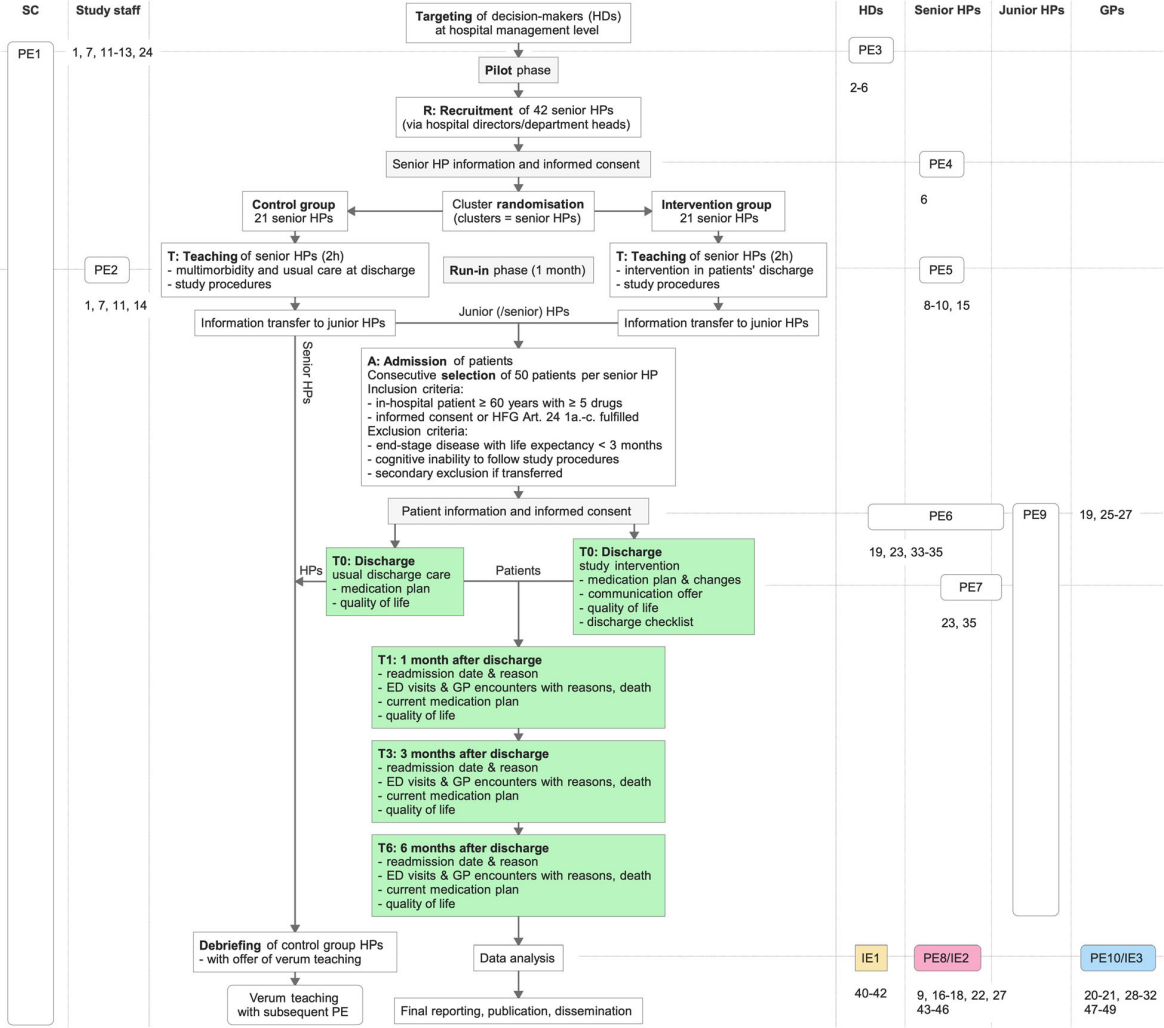
Study flow chart with accompanying process and impact evaluation. Coloured rectangles provide data for the impact evaluation (IE), and rounded edges indicate stages of the process evaluation (PE1-10). Numbers refer to the items in Additional file 1. SC, study centre; HD, hospital director; HP, hospital physician; GP, general practitioner

The core trial has been designed as a prospective, double-blind, bi-centre, cluster-randomised parallel-controlled study with 4 months of patient recruitment per cluster and individual follow-ups of 6 months and will be accompanied by a process evaluation study (see Fig. 1 for the study flow chart).

### Study population

Patients in hospitals of all types, levels and legal structures in northern, eastern and central Switzerland are eligible and can participate if they fulfil all the following inclusion criteria:

- In-hospital patient at the time of inclusion
- Male or female of 60 years or older with five or more drugs prescribed
- Signed informed consent or—in case of a patient incapable of judgement—consent from a legal representative according to Swiss law (HRA Art. 24, 1a.-c. and SCC Art. 378)

The prescription of five or more drugs (a commonly used definition of polypharmacy [26]) is used as a proxy for multimorbidity, and patients with cognitive impairment are included as well in order to increase generalisability of the results.

The following exclusion criteria will apply:

- End-stage disease with a life expectancy of less than 3 months
- Cognitive inability to follow study procedures neither independently nor with assistance
- Secondary exclusion: patient is transferred to another ward or another hospital

Hospitals which took part in the Swiss national pilot project “progress! Sichere Medikation an Schnittstellen” [27] will not be considered for participation in the study because the said project involved a medication review which might confound our intervention effect.

### Recruitment and allocation

Eligible hospitals will be contacted on the medical management level with detailed written information about the study and an invitation to participate. Each consenting senior HP from a participating hospital, together with his ward’s junior HPs and their patients, will constitute a cluster. Choosing disjoint clusters will minimise the possibility of contamination within the same hospital ward.

Sufficiently large blocks of clusters [28] will be allocated to the study arms using covariate-constrained randomisation [29], i.e. by randomly choosing (by an independent third party) from a set of randomly generated allocation schemes with sufficient balance in terms of hospital types (acute-care vs rehabilitation, rural vs central, academic vs non-academic) as well as type (medical discipline) and size (number of beds) of hospital wards.

In-hospital recruitment of patients will be performed by HPs or nurses; a person responsible for recruitment will be defined in every ward. Patients will be recruited during 4 months or until the targeted number of 50 participants per cluster is reached.

### Blinding

Blinding of HPs in its strictest sense is not possible within the chosen study design. In order to achieve a certain degree of blinding, the HPs will be informed that the study aims at investigating the effects of different discharge strategies on readmission times but they will not be offered detailed information about the other study arm. This will prevent the discharging HPs and their patients from knowing which of the two arms they were allocated to.

### Intervention

The intervention will take place on different levels with different target populations:

#### A. Cluster level: “Teach-the-Teachers” session for the senior HPs in charge of postgraduate training and supervision of the junior HPs

The purpose of this training of 2 h duration is to teach and motivate HPs to integrate a specific discharge procedure (Table 1) into the daily work of the HPs. The teaching session will address the following items:

- How to identify eligible patients according to the in- and exclusion criteria
- How to apply a simple medication review tool to the patients’ medication list
- How to involve the senior HPs in the medication reviews
- How to identify the patients’ needs and priorities
- How to involve the patient in active shared decision making about his/her treatment
- How to create revised discharge medication plans for the patients
- How to involve the GPs in the post-discharge period
- How to deal with the different data collection forms

**Table 1:** Checklist for the discharging hospital physician

	Yes	No
1: Have you collected the main complaint of the patient?	□	□
2: Have you and your patient discussed the treatment goals from his own point of view?	□	□
3: Have you compiled a full list of all the patient’s drugs at admission?	□	□
4: Have you decided for every single drug whether		
▪ the patient will indeed take it as prescribed?	□	□
▪ the indication of the drug is correct for this patient?	□	□
▪ the risk of side effects (present or expected) is less than the benefit incurred?	□	□
▪ the dose is correct for this individual patient (age, comorbidities)?	□	□
▪ there is no alternative drug with a better benefit-to-risk ratio?	□	□
5: Have you decided whether a new drug is indicated?	□	□
6: Did you involve the patient in the changes you are proposing?	□	□
7: Have you provided the patient with a discharge medication list together with an invitation to use it?	□	□
8: Have you motivated the patient to consult the family doctor/general practitioner within 7 days?	□	□
9: Did you send the list of modified or newly introduced medications to the family doctor/general practitioner?	□	□
10: Was there any contact with the general practitioner during the hospital stay in view of the imminent discharge of the patient?	□	□

Following their own training, the senior HPs will instruct the junior HPs how to apply the structured discharge procedure to their patients. The senior HPs will also be responsible for the instruction of junior HPs who get newly assigned to their wards as part of the junior HPs’ job rotations. This approach guarantees consistency of the intervention even where fluctuation among junior HPs is high.

#### B. Patient level: The intervention at discharge

The junior HPs will perform critical reviews of their patients’ medication lists, supervised by their senior HPs, discuss the results of these reviews and their suggestions with the patients and create optimised discharge medication plans to be used by the patients. To ensure correct and complete implementation of the discharge procedure, the junior HPs will document these steps by ticking answer boxes on the specific checklist (Table 1). The patients will be encouraged, by written and direct verbal information, to consult their GPs within 7 days of being discharged.

Depending on features and flexibility of the hospital information system and its electronic record features, the junior HPs will communicate either the final revised medication lists or listings of all medication changes (cessation of specific medication, change of dosage, introduction of new drugs) to the patients’ GPs in written form. Furthermore, these notifications will be accompanied by invitations to the GPs to discuss the medication changes.

In the control arm, the senior HPs will undergo a 2 h educative session addressing multimorbidity, the need for epidemiological and outcome data for this specific population, patient in- and exclusion criteria and the handling of the different data collection forms. All patients in the control group will be discharged according to the usual local routines.

### Implementation

**Fig. 2:**
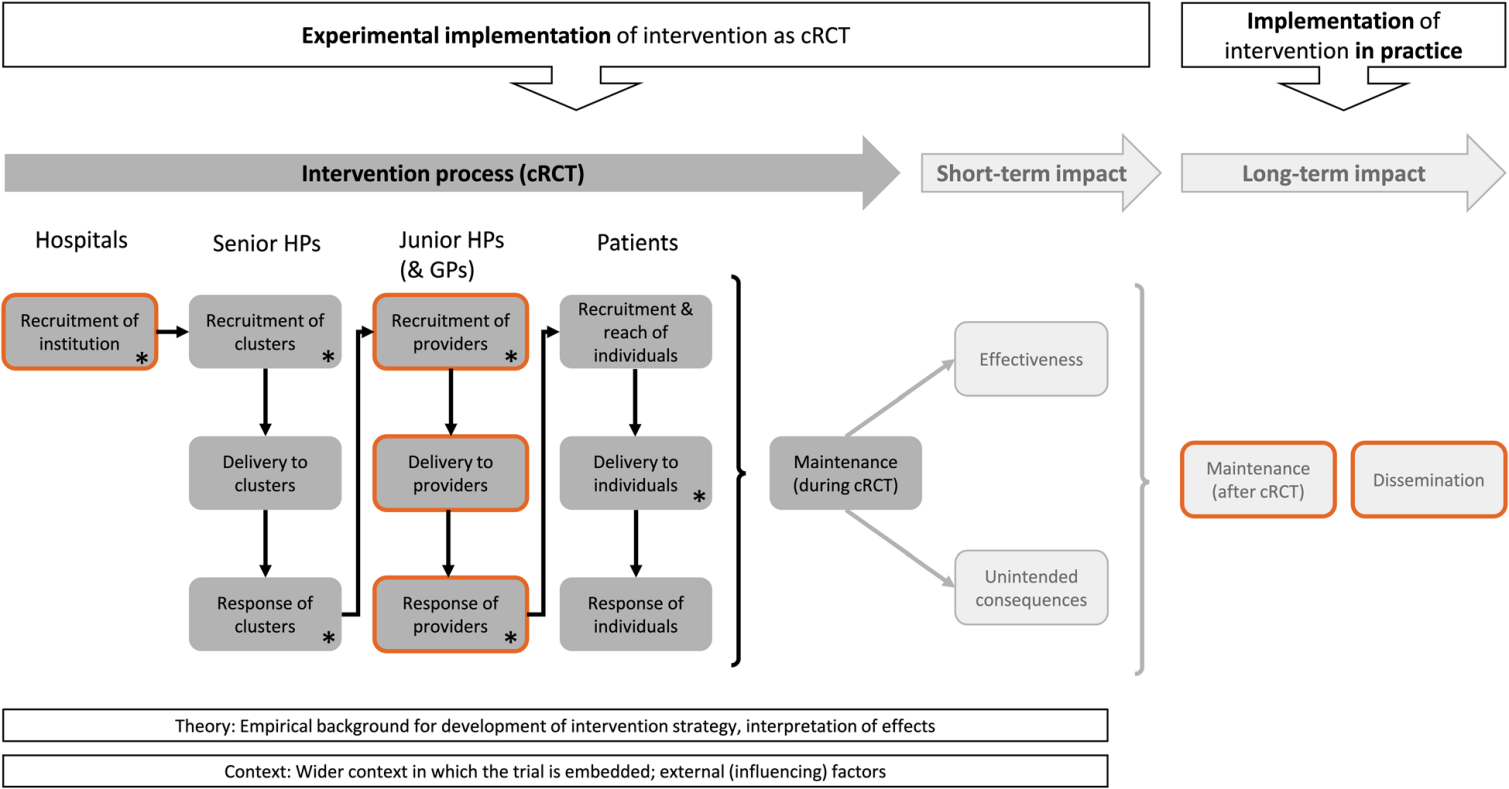
Framework model for process (dark grey) and impact evaluation (light grey), adapted from Grant et al. [32]. Bold frames indicate extensions to the original framework; asterisks mark thematic focus. cRCT, cluster RCT; HP, hospital physician; GP, general practitioner

The implementation of the discharge strategy under study can be understood as a two-step process as illustrated in our model in Fig. 2. Step 1 represents the experimental implementation of the intervention as a cluster RCT, covering the intervention process itself as well as the short-term impact of the intervention, while step 2 constitutes the implementation of the intervention in practice, comprising maintenance and dissemination (long-term impact).

### Outcome measures

The primary and secondary outcome measures will be collected on the individual patient level from hospital records or from the patients themselves (patient records or questionnaires).

#### A. Primary outcome

Number of days until the first readmission to (any) hospital within 6 months after discharge.

#### B. Secondary outcomes

- Readmission rates at 1, 3, and 6 months after discharge
- Number of emergency department (ED) visits or GP encounters within 1, 3 and 6 months after discharge
- Death during follow-up of 6 months
- If applicable: reasons for readmission, ED visits, GP encounters or death
- Number of drugs at discharge and at 1, 3, and 6 months after discharge
- Anatomical Therapeutic Chemical (ATC) classes of the drugs prescribed/deprescribed at discharge and at 1, 3, and 6 months after discharge
- Proportion of potentially inappropriate medications (PIMs) based on 2012 Beers criteria [30] and the PRISCUS list [13] at discharge and at 1, 3, and 6 months after discharge
- Patients’ quality of life on the EQ-5D-3L-scale [31] at discharge and at 1, 3, and 6 months after discharge

The following covariates will be collected at the time of inclusion of clusters or individuals, respectively, and used for balanced cluster allocation and subgroup analyses:

- Patient characteristics (age, sex)
- Hospital type (acute-care, rehabilitation, rural, central, academic, non-academic)
- Type (medical discipline) and size (number of beds) of hospital wards

#### C. Process and additional impact outcomes

At several stages of the ongoing RCT, additional process and impact outcomes will be collected from all relevant stakeholders (Fig. 1 and Additional file 1). The evaluation will follow an adapted form of the framework proposed by Grant et al. [32] (Fig. 2).

In its original form, the framework has been developed for two-levelled cluster RCTs of complex interventions, addressing clusters (in the present study: senior HPs) and individuals (here: patients). In order to account for the hierarchical structure of our study, we added two additional levels to the framework (marked with orange frames in Fig. 2) representing the institutions (hospitals) and direct providers (junior HPs), respectively, thus resulting in a four-dimensional model of the intervention process (Fig. 2).

Additional file 1 contains a comprehensive list of items addressed within the process and impact evaluation study, with mappings to their respective framework elements, target groups and study stages.

##### a. Process

The process evaluation will cover the recruitment of the institutions as well as recruitment, intervention delivery and response with regard to all other cluster hierarchy levels. Generally, we will describe the implementation together with facilitating strategies as carried out but also characteristics, exposure and the experiences of the target groups, as suggested in [33–35]. Thereby, we intend to identify mechanisms that potentially act on primary and secondary outcomes and to explore factors affecting the implementation process including implementation fidelity [36]. Accordingly, we will focus (indicated by * in Fig. 2) on the recruitment of hospitals and health professionals, including the description of their characteristics, as well as on their response and the actual intervention delivery to the patients.

##### b. Impact

Short-term: The assessment of the effectiveness of the intervention on the patient-level, i.e. the effects on primary and secondary outcomes [32], is part of the core RCT and has been described above. Additionally, we will record unintended consequences of the intervention, meaning “change in other outcomes which may be perverse, harmful or beneficial” [32].

Long-term: Since—in case of positive outcomes—we intend to assess and promote long-term maintenance of the intervention in recruited hospitals after completion of the trial, and to foster potential distribution to further hospitals, we included the assessment of the long-term impact of the intervention subsequent to the cluster RCT in our framework model.

##### c. Empirical background and context

In addition to the cluster structure of the trial, the framework we adapted for process and impact evaluation also considers the empirical basis underlying the development of the intervention as well as the context in which the trial is being conducted, the latter also with regard to assessing the generalisability of the study results.

Evaluating the effectiveness of the intervention will allow inference on the empirical background (see the Background section above) and its adequacy for the study design. Context factors to be studied include characteristics of the hospitals (spectrum of medical services offered, diversity and flexibility of patient information systems, financial and other resources available for innovation and research, etc.) and of the follow-up GPs and their communication with the discharging HPs (eg. perception of the communication offers, frequency of communication, reasons to contact the discharging HPs).

##### d. Operationalisation

The list in Additional file 1 operationalises the concept outlined above. For example, ratings of feasibility and acceptance of the intervention will be collected in order to assess its relevance from both senior and junior HPs (by items 16 and 25) using 5-point Likert scales (Additional file 2). Barriers to and enablers of deprescribing will be explored among senior HPs in telephone interviews (item 18) following a qualitative method approach with both Likert items and open-ended questions (Additional file 3). Context factors pertaining to the hospitals and hospital directors will be collected in item 6, and in order to measure the adequacy of the communication triggers, item 27 will capture HP-GP contacts during inpatient stays with regard to the impending discharge, as well as the frequency of the GPs’ utilisation of communication offers from HPs.

##### e. Ethics

For ethical reasons, the control group hospital directors and HPs will get full access to the intervention (training for the deprescribing intervention at discharge) after completing the study. Consecutively, their process outcome measures will be explored as well, similar to the intervention group exploration as described above.

### Follow-up

One, 3, and 6 months after discharge, the patients will be contacted in writing by the study centre and asked to report any readmissions, ED visits and GP encounters (with reasons, if applicable) since discharge in paper case report forms (CRFs). The patients will also be asked to submit their current medication plans and short QoL questionnaires to the study centre. In case of outstanding responses, the study team will contact patients, relatives, GPs, and/or hospitals by phone or in written form in order to complete the missing data.

### Data collection procedures

The senior HPs will document the participation of each patient in an enrolment log to be kept at their hospitals. The study centre will keep a file of CRFs for each study participant with all relevant data pertaining to the participant during the study. The forms will be encoded, and the codes will be stored at the hospitals. Decoding will be possible if case-tracking is needed (in case of adverse events). For coding, data and query management, monitoring and reporting purposes, the current version of the clinical data management tool “OpenClinica” (OpenClinica, LLC) will be used. The transfer from paper to electronic data will be carried out and independently double-checked by different research associates.

### Sample size calculation

We modelled hospital readmission time by fitting an exponential survival curve on published readmission rates [20, 22] and internal data from the University Hospital Zurich. Extending readmission time by 25% [20] was considered a relevant effect of the intervention. In the fitted model, this translates into an increase of the median readmission time by 23.2 days (from 92.7 days to 116.8 days) and corresponds to a hazard ratio of 0.80 between intervention and control group participants. This is equivalent to a decrease of 18.2% in readmission rates after 30 days (from 20.1% to 16.4%).

Based on this hazard ratio and assuming a two-sided α = 5%, a power of 1-β = 80%, an intraclass correlation coefficient (ICC) of 0.02 as in [37], 40% overall censoring probability and equally sized clusters of 50 patients, we calculated a sample size of 21 clusters and 1050 individuals per trial arm. Thus, 42 senior HPs and 2100 patients in total will need to be included in order to observe a relevant effect of the study intervention with sufficient power.

### Plan of statistical analyses

Descriptive statistical methods will be used to describe the study population, including dropouts and losses to follow-up. Baseline characteristics of both intervention and control group will be calculated with corresponding 95% confidence intervals where applicable. In particular, baseline variability among different hospitals will be assessed by retrospectively analysing the discharge prescriptions of all includable patients in the last month prior to the start of patient recruitment.

The primary outcome will be compared between groups using Kaplan-Meier estimators and log-rank tests. To compare factors which may affect readmission, the Cox proportional hazards model will be used considering clustering by senior HPs as a random factor. The multivariable model will include patient characteristics as well as all covariates used for balanced allocation, and subgroup analyses may be carried out for such factors or covariates.

An interim analysis is planned once 50% of the participants have been recruited.

For secondary outcomes, parametric (*t* test) or non-parametric tests (*χ*^2^ and Wilcoxon tests) will be used as appropriate. Determinants associated with a change of the medication will be investigated by exploratory, multivariate regression analysis.

The analyses of primary and secondary outcomes will follow the intention-to-treat principle. Missing values will be replaced by standard multiple imputation (MI) as recommended by Ma et al. [38] for clustered designs with variance inflation factors < 3. Per-protocol analyses will be performed within the scope of sensitivity analyses.

As a secondary analysis using the original data, a cost analysis will be carried out on the basis of present medication prizes, and costs saved by spared drugs and avoided hospitalisations will be estimated.

For process evaluation purposes, a qualitative methods approach will be adopted to analyse data from questionnaires and interviews (either face to face or by phone). Ratings of feasibility and acceptance of the intervention (Additional file 2) and quantitative data gained from the exploration of barriers and enablers of deprescribing (Additional file 3) will be summarised and presented using descriptive statistics and graphical methods. For the interviews with study staff, a focus group of hospital directors, senior HPs and with a random subset of GPs, semi-structured interview guides will be used with room for open-ended comments (an example is given in Additional file 3.) The structured part of the interview will cover key areas compiled from the relevant literature [39–45] by consensus among the study team, while the open part will be evaluated using quantitative content analysis methodology [46, 47]. In a first inductive step, the answers will be searched for codes not fitting into the predefined key areas. New key areas will be defined accordingly, again by consensus, until saturation is reached. In the second step, all open-ended answers will be coded using the refined list of key topics.

### Timeframe

**Fig. 3:**
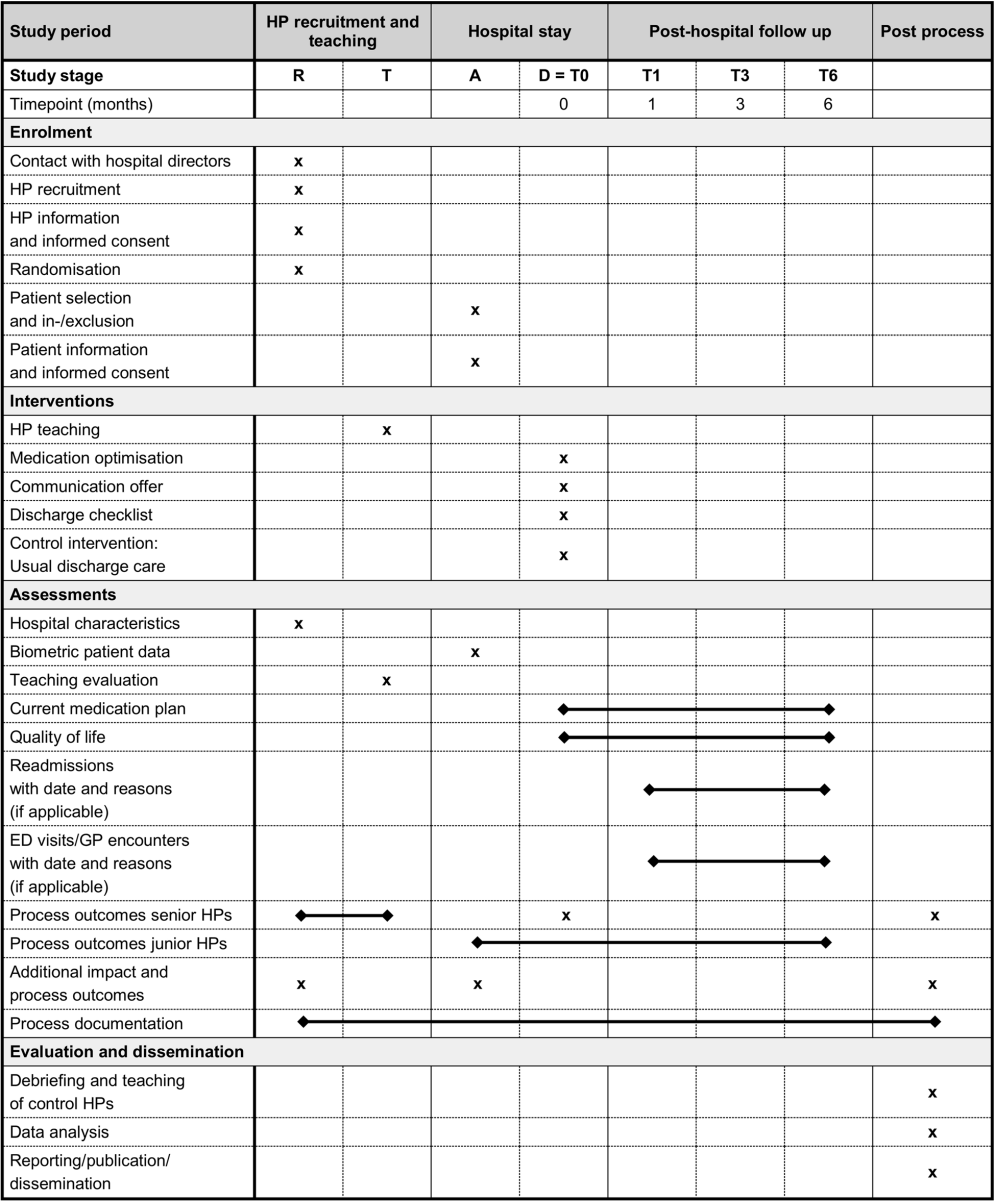
Study schedule (SPIRIT diagram of trial stages of enrolment, intervention, outcome assessment and evaluation). R, Recruitment; T, Training; A, Admission; D, Discharge; T1/3/6, follow-ups at 1/3/6 months after discharge; HP, hospital physician; ED, emergency department; GP, general practitioner

The study will start with a short pilot test phase to check the study tools in late 2018. Searching for participating hospitals and recruitment of senior HPs will take place between mid- and end of 2018, and inclusion of the first patients is planned for early 2019. For more details about the study schedule, see the SPIRIT diagram (Fig. 3):

### Patient safety and monitoring

The study is considered to entail only minimal risks and burdens for the medical personnel and patients involved since the intervention consists of education and—on the patient level—critical reviews and potential optimisations of discharge medication plans by trained physicians with the possibility to immediately reverse any change in case of unintended side effects. Therefore, and because the retrospective collection of safety outcomes (readmissions, ED visits, GP encounters, deaths) forms an integral part of the study design, no dedicated safety board will be established. Possible causal dependencies of serious adverse events on the study intervention will be assessed by the study team and reported to the responsible ethics committee. Insurance is covered by the University Hospital Zurich’s open policy for clinical and non-clinical trials.

The study centre will collaborate with the Clinical Trials Center (CTC) of the University Hospital Zurich to ensure monitoring. All original data including the patient files (in particular all written informed consents and the CRFs) will be subject for monitoring. Monitoring will be performed primarily by phone and—if needed—by visits at the project sites.

### Confidentiality and data security

The patient names and all other confidential information fall under medical confidentiality rules and will be treated according to applicable Swiss data security laws. For contact maintenance and case tracking (e.g. in case of adverse events), the patients’ identities will be known to a study nurse not involved in the analysis of the patient data. The patients’ names will not be accessible to the scientific study staff. All electronic data, including interview transcripts, will be stored under password protection on secure network drives of the University Hospital Zurich.

## Discussion

### Study rationale

Several recently published protocols describe trials which aim at examining the effects of individual medication reviews taking the patients’ views on medication appropriateness and treatment priorities into account [48, 49]. Our study pursues similar goals but is unique with respect to, firstly, the setting at hospital discharge as a crucial interface between HPs and GPs, and secondly, the addition of a communication strategy to the medication review. By inviting the follow-up GPs to discuss the prescribing/deprescribing decisions, we hope to achieve a better consensus between HPs, GPs, and patients on their medication plans, and thus a lower rate of switching back to prior medication lists and a higher rate of patient adherence to their medication plans.

### Strengths and limitations

Strengths of this study include its focus on the interface between hospital medicine and primary care, its flexible set-up regarding pre-existing in-hospital structures and the investigation of both the intervention tool itself as well as its implementation.

The transition from hospital back to home is a delicate but crucial step on the patients’ paths to resume their usual lives. To our knowledge, the study intervention is unique in its objective to structure and organise this step, not only by providing a set of instructions regarding optimal discharge medication but–beyond that–by implementing communication enablers between all actors involved.

The study design is flexible in so far as in order to minimise the additional workload of the participating HPs, optimal data collection and transmission procedures at discharge will be worked out individually and cooperatively with each hospital or hospital ward.

The accompanying process and implementation evaluation will contribute to a better explanation of the intervention effects and provide valuable information needed for potential optimisation of the discharge strategy under study and later propagation and dissemination in larger settings.

A possible limitation might be that the contamination between clusters within the same hospital cannot be fully excluded. Varying degrees of protocol adherence by the discharging HPs in the intervention arm and considerable inhomogeneity among the discharge routines followed by different hospitals in the control arm of the study might partly obscure the intervention effect.

### Conclusion

The results of the present study will enlarge the knowledge base about optimal discharge procedures of elderly multimorbid patients. If successful and well received, the intervention under study has the potential to propel the development of better discharge routines and may fuel initiatives to reduce polypharmacy and its associated adverse effects among discharged patients. Ultimately, the study could lead to both cost reductions in the health care system and gains in quality of life for patients.

From the process assessment and implementation results, we expect to learn whether our approach will be accepted in Swiss hospitals of different provenance and if it has the potential to become a best clinical practice model for hospital discharge and medication management in the future [50].

### Trial status

Patient recruitment has not yet started at the time of the first submission in September 2018 and is planned to start in early 2019 and last until mid-2019.

## Additional files

Additional file 1: List of PE/IE items. (PDF 16 KB)

Additional file 2: CRF feasibility and acceptance. (PDF 47 KB)

Additional file 3: Interview guide for senior HPs (barriers and enablers). (PDF 62 KB)

## Abbreviations

ATC: Anatomical Therapeutic Chemical classification system
CI: Confidence interval
CTC: Clinical Trials Center
ED: Emergency department
GP: General practitioner
HP: Hospital physician
HRA: Human Research Act
MI: Multiple imputation
PIM: Potentially inappropriate medication
QoL: Quality of life
RR: Relative risk
SCC: Swiss Civil Code
