# Supplementary material for: Improving inappropriate medication and information transfer at hospital discharge: study protocol for a cluster RCT
Source: Implement Sci. 2018 Dec 27;13:155. doi: 10.1186/s13012-018-0839-1 (PMC6309068; doi:10.1186/s13012-018-0839-1)
Supplement: Supplementary file 1 — List of PE/IE items. (PDF 16 KB) [file 13012_2018_839_MOESM1_ESM.pdf]

No	Elements of process and impact evaluation and corresponding items for evaluation	Target group							Study stage													
		SC	SSt	HoD	sHPs	jHPs	GPs	Pat	PE1	PE2	PE3	PE4	PE5	PE6	PE7	PE8	PE9	PE10	OCs	IE1	IE2	IE3
	**PROCESS EVALUATION**																					
	**Recruitment of institution**																					
1	Which procedure has been adopted to recruit the hospital directors (first contact with hospitals, information for hospital directors, reminders, waiting lists)? What has proven itself?	X	X						X	X												
2	How do the hospital directors perceive/assess the information offered about the study?			X							X											
3	What expectations, concerns, and motivation do the hospital directors harbour regarding the intervention (e.g., applicability, usefulness)?			X							X											
4	What is the study's degree of acceptance among the hospital directors (e.g. regarding the role of the Institute of Primary Care in the hospital context)?			X							X											
5	What reasons are given by hospital directors refusing to participate?			X							X											
6	Which institutional and organizational factors favour or hamper the implementation of the intervention (Characteristics of hospitals (covariates), e.g. type of hospital, size of department or ward, presence or absence of quality manager)?			X	X						X	X										
	**Recruitment of cluster**																					
7	Which procedure has been adopted to recruit the senior HPs (e.g. contact, information)? What has proven itself?	X	X						X	X												
8	How do the senior HPs perceive/asses the information offered about the study?				X								X									
9	Which senior HPs participate in the training (individual characteristics, attitudes)?				X								X			X						
10	What is the motivation of the senior HPs to take part in the training or to carry out the intervention (reasons for participating in the study)?				X								X									
	**Delivery to cluster**																					
11	How were the teachings for the senior HPs designed and carried out (e.g., training materials, number of training sessions, duration, instructor)? What has proven itself?	X	X						X	X												
12	What support do the senior and junior HPs receive for implementing the intervention and communicating with the GPs (e.g. recruitment criteria, documentation, tools, contact person/opportunities in case of questions)?	X							X													
	**Response of cluster**																					
13	To what extent and purpose do the senior HPs use the opportunities to ask questions (number and content of inquiries)?	X							X													
14	Based on the inquiries received, are there indications of potential for improvement regarding information and training of the senior or junior HPs?	X								X												
15	How satisfied are the senior HPs with the training overall (e.g. content, quality, benefit, effort)? Are all needs and expectations covered?				X								X									
16	How do the senior HPs perceive the intervention approach (e.g., applicability, benefits gained and effort required, acceptance)?				X											X						
17	How do the senior HPs assess the support they receive from the Institute of Primary Care for the implementation (documents, instruments)?				X											X						
18	How do the senior HPs rate their overall experience with implementing the intervention (e.g. difficulties, benefits, effort, contacts with GPs)?				X											X						
	**Recruitment of providers**																					
19	What are the attitudes of the participating junior HPs toward the reduction/optimisation of medication lists during patient discharge?					X								X			X					
20	According to the GPs, how relevant is the possibility to discuss medication plans of their patients with the discharging HPs?						X											X				
21	What are the attitudes of the GPs toward the reduction/optimisation of medication lists during patient discharge?						X											X				
	**Delivery to providers**																					
22	How do the senior HPs handle the instruction of the junior HPs?				X											X						
23	How do the senior HPs implement the intervention in the hospital? To what extent and for what reasons do deviations from was the planned implementation occur?				X									X	X							
	**Response of provider**																					
24	To what extent and purpose do the junior HPs use the opportunities to ask questions (number and content of inquiries)?	X							X													
25	How do the junior HPs perceive the intervention approach (e.g., applicability, benefits gained and effort required, acceptance)?					X											X					
26	How do the junior HPs assess the support they receive from the Institute of Primary Care and from the senior HPs for the implementation (documents, instruments, instruction and support from senior HPs)?					X											X					
27	How can the contacts/interactions between the discharging HPs and the GPs be described (e.g. number, duration)? Do the GPs contact the senior or the junior HPs?					X	X									X	X					
28	How do the GPs perceive the communication opportunities with the discharging HPs (benefits gained, effort required, acceptance)?						X											X				
29	What are the reasons for GPs to either continue to use or re-adjust their patients' customised discharge medication plans?						X											X				
30	For what reasons do the GPs contact the discharging HPs?						X											X				
31	How do the GPs rate the information from the HPs about the medication plans?						X											X				
32	How do the GPs assess their overall experience with the contacts to the discharging HPs?						X											X				
	**Recruitment of patients**																					
33	Which procedure has been adopted for patient recruitment (e.g. application of recruitment criteria, information material, recruitment staff)?			(X)	X	X								X								
34	Which patients participate in the study (age, sex)?							X						X								
	**Delivery to patients**																					
35	How do the junior HPs implement the intervention in the hospital? To what extent and for what reasons do deviations from was the planned implementation occur?					X								X	X							
	**Response of patients**																					
36	How do the patients experience the intervention and what is their response?							X								X	X					
	**IMPACT EVALUATION**																					
	**Maintenance (during cluster RCT)**																					
37	Is the intervention delivered consistently over the course of the study?																					
	**Effectiveness**																					
38	See primary and secondary outcome measures as described in the main article							X											X			
	**Unintended consequences**																					
39	Did changes in other outcomes occur?	X	X	X	X	X	X	X	X													
	**Long-term maintenance**																					
40	From the hospital directors' perspective, are there any improvements in their hospitals due to the participation in the study?			X																X		
41	What are the hospital directors' estimates of the potential for cost savings as a result of the intervention?			X																X		
42	From the hospital directors' point of view, in what ways does the approach prove successful in terms of further dissemination in Swiss hospitals? Which stakeholders should be involved according to the hospital directors in order to disseminate the approach?			X																X		
43	From the senior HPs perspective, are there any improvements in their hospitals due to the participation in the study?				X																X	
44	From the senior HPs point of view, has the communication with the GPs improved due to the participation in the study?				X																X	
45	To what extent do the senior HPs continue to follow the approach after the study? Which factors contribute to this?				X																X	
46	In what ways do the senior HPs intend to recommend the approach to their colleagues?				X																X	
47	Do the GPs still receive invitations from the discharging HPs to discuss discharge medication plans after the end of the study?						X															X
48	After termination of the study, do the GPs continue to contact the HPs in order to discuss the discharge medication plans of their patients?						X															X
49	To what extent is the approach suitable for further dissemination from the GPs point of view?						X															X

Legend: SC: Study center; SSt: Study staff; HoD: Hospital directors; sHPs: Senior hospital physicians; jHPs: Junior hospital physicians; GPs: General practicioners; Pat: Patients; PE: Process evalualtion; OCs: Outcome measures; IE: Impact evaluation
