# Supplementary material for: Improving inappropriate medication and information transfer at hospital discharge: study protocol for a cluster RCT
Source: Implement Sci. 2018 Dec 27;13:155. doi: 10.1186/s13012-018-0839-1 (PMC6309068; doi:10.1186/s13012-018-0839-1)
Supplement: Supplementary file 2 — CRF feasibility and acceptance. (PDF 47 KB) [file 13012_2018_839_MOESM2_ESM.pdf]

Universität Zürich[UZH]

Institut für Hausarztmedizin

Hospital Discharge Study

UniversitätsSpital Zürich

Feasibility and acceptance

Cluster-ID: …………….

Dear colleague

We would like to ask for your assessment of the feasibility and acceptance of the investigated discharge model. Please answer the following questions:

- The discharge checklist is well suited for daily work.

○	○	○	○	○
1= strongly disagree	2	3	4	5 = fully agree

- The discharge checklist is helpful for optimising the medication.

○	○	○	○	○
1= strongly disagree	2	3	4	5 = fully agree

- I will continue to use the discharge checklist after the end of the study.

○	○	○	○	○
1= strongly disagree	2	3	4	5 = fully agree

- I would recommend the discharge checklist to my colleagues.

○	○	○	○	○
1= strongly disagree	2	3	4	5 = fully agree

- My patients reacted positively to the discharge procedure.

○	○	○	○	○
1= strongly disagree	2	3	4	5 = fully agree

Thank you very much for your highly valued cooperation!

Please send the completed form **along with the originals of all inclusion lists** to the study centre.
Thank you!
