# Supplementary material for: Improving inappropriate medication and information transfer at hospital discharge: study protocol for a cluster RCT
Source: Implement Sci. 2018 Dec 27;13:155. doi: 10.1186/s13012-018-0839-1 (PMC6309068; doi:10.1186/s13012-018-0839-1)
Supplement: Supplementary file 3 — Interview guide for senior HPs (barriers and enablers). (PDF 62 KB) [file 13012_2018_839_MOESM3_ESM.pdf]

Universität Zürich[UZH]

Institut für Hausarztmedizin

Hospital Discharge Study

UniversitätsSpital Zürich

Telephone interview guide for senior HPs

Cluster-ID: …………….

«Grüezi» Doctor ………….……….…….....…….…, my name is ……………..……..……..….…… and I'm with the Institute of Primary Care of the University of Zurich. In the past year, you participated in our Hospital Discharge Study about optimising medication and communication at hospital discharge of multimorbid elderly patients. Thank you again for your participation!

I would like to ask you some final questions. Can you spare just a few minutes, or would you prefer me to call again at another time?

(If applicable, date and time of scheduled interview: ……………………..…………………….....…………….)

I will ask you a few questions about your experience with the study and about possible barriers that you may have encountered or ways in which the implementation of the intervention could be facilitated. Our conversation should last for 10 minutes at most.

Most questions will need to be answered on rating scales with levels 1-5, with 1 meaning strong disagreement and 5 meaning total agreement. There will be some open-ended questions, too. If you have any questions, please interrupt me at any time.

Opening questions

1. Overall, how would you gauge your own and your junior colleagues' experiences with counselling patients on their optimal medication plans?

○	○	○
1= negative	3 = neutral	5 = positive

In what ways? Why? …………………………………………………………………………………………………….

2. Would you or your junior physicians like to discuss their medication with your patients more frequently or more thoroughly (e.g. talk about how to shorten the medication lists)?

○	○	○	○	○
1= strongly disagree	2	3	4	5 = fully agree

3. Would you welcome the introduction of a systematic medication review at hospital discharge?

○	○	○	○	○
1= strongly disagree	2	3	4	5 = fully agree

Enablers

4. In your opinion, what would facilitate the introduction of a systematic medication review at hospital discharge (in general and for you personally)?

…………………..……………………………………………………………………………….…………………….

5. Did your patients appreciate the opportunity to talk and decide about their own medication lists (in the sense of shared decision making)?

O	O	O	O	O
1= strongly disagree	2	3	4	5 = fully agree

Comments: ……………………………………………………………………………………………………….

6. Were there any groups or types of patients for whom it was either particularly easy or more difficult to omit certain medications (e.g. personality traits of patients or disease constellations)?

O	O	O	O	O
1= strongly disagree	2	3	4	5 = fully agree

Which ones? Why? ………………………………………………………………………………………………

7. How satisfied were the patients with the remaining set and number of medications?

O	O	O	O	O
1= very dissatisfied	2	3	4	5 = very satisfied

Why? Too many, too few? Good or bad experiences? Too many changes? ……..……..……..…………..…….

……………………..……………………………………………………………………………………………….

8. Did you have the impression that the patients still felt being taken seriously even when a drug was «taken away» (role of the doctor - caring)?

O	O	O	O	O
1= strongly disagree	2	3	4	5 = fully agree

Comments: ……………...………..…………………………………………………………………………….

Barriers

9. Did the patients have reservations about the proposals of the discharging physicians?

O	O	O	O	O
1= strongly disagree	2	3	4	5 = fully agree

For what reasons or with what fears and about what proposals in particular? …………………………………..

……………………..……………………………………………………………………………………………….

10. In your opinion, did the patients have the feeling that they were deprived of something well-tried and proven (their accustomed medication)?

O	O	O	O	O
1= strongly disagree	2	3	4	5 = fully agree

If 1 or 2: For how long, approximately, had they been taking these medicines? ……………………..…..……...

……..

11. Did you or your assistant physicians have inhibitions or reservations about changing established medication lists?

O	O	O	O	O
1= strongly disagree	2	3	4	5 = fully agree

Reasons? In which constellations? With any particular drugs? ………………………..………………………

……..

12. Did you or your junior physicians have to overcome internal resistance when carrying out a systematic medication review?

O	O	O	O	O
1= strongly disagree	2	3	4	5 = fully agree

Why? Time constraints? Fears? Doubts about purpose and benefit? …………………………………………...

……..

Concluding questions

13. Would you like mention anything else about your experiences with deprescribing, or add any thoughts related to our study?

………......

………......

………......

14. Do you have any questions for me? If not, thank you very much for your precious time and cooperation.

Date: Interviewer:

……………………………………………. ………………………………………………………………………………
